# Circ_0087851 suppresses colorectal cancer malignant progression through triggering miR-593-3p/BAP1-mediated ferroptosis

**DOI:** 10.1007/s00432-024-05643-3

**Published:** 2024-04-20

**Authors:** Ming Huang, Ting Gao, Xianyong Chen, Junlei Yi, Xuan Zhou

**Affiliations:** 1 Department of Pathology, Affiliated Hospital of Xiangnan University, Chenzhou, 423000 Hunan China; 2 Gastrointestinal Surgery, Affiliated Hospital of Xiangnan University, No. 25, Renmin West Road, Beihu District, Chenzhou, 423000 Hunan China

**Keywords:** Colorectal cancer, Circ_0087851, MiR-593-3p, BAP1, Ferroptosis

## Abstract

**Background:**

Emerging research has validated that circular RNAs (circRNAs) have indispensable regulatory functions in tumorigenesis, including colorectal cancer (CRC). Ferroptosis is a specific cell death form and implicates in the malignant progression of tumors. Here, this study aimed to investigate the biofunction of circ_0087851 in tumor progression and ferroptosis of CRC, as well as its underlying molecular mechanism.

**Methods:**

The expression pattern of circ_0087851 in CRC was validated by qRT-PCR. The biological characteristics of circ_0087851 in CRC were assessed through CCK-8, colony formation and transwell assays in vitro. The ferroptosis was measured using ferroptosis-related reagents on iron, Fe^2+^, and lipid ROS detection. Bioinformatics, luciferase reporter, and RNA pulldown assays were employed to reveal the circ_0087851-mediated regulatory network. In addition, the effect of circ_0087851 on tumor growth in vivo was detected using a xenograft model.

**Results:**

Circ_0087851 was notably diminished in CRC tissues and cells. Functionally, overexpression of circ_0087851 suppressed CRC cell growth, migration, invasion, and facilitated ferroptosis in vitro. Meanwhile, circ_0087851 upregulation impeded CRC growth in vivo. Mechanistically, circ_0087851 functioned as a molecular sponge for miR-593-3p, and BRCA1 associated protein 1 (BAP1) was identified as a downstream target of miR-593-3p. Besides, rescue experiments revealed that miR-593-3p overexpression or silencing of BAP1 reversed circ_0087851-mediated CRC progression.

**Conclusion:**

Circ_0087851 performed as a tumor suppressor and ferroptosis promoter by the miR-593-3p/BAP1 axis, providing novel biomarker and therapeutic target for the clinical management of CRC.

**Supplementary Information:**

The online version contains supplementary material available at 10.1007/s00432-024-05643-3.

## Introduction

Colorectal cancer (CRC) is a common malignancy worldwide that causes tremendous damage to human health. According to Oncology statistics, CRC has the third highest incidence rate globally and is also the second leading cause of cancer-related deaths (Sung et al. 2021). Diet, lifestyle habits (Puzzono et al. 2022), and genetic factors (Patel et al. 2022) are inextricably involved in the emergence and advancement of CRC. Although a variety of treatments have been widely employed in clinical management of CRC, including surgery, chemotherapy, and immunotherapy, the therapeutic benefit of CRC remains unsatisfactory (Biller and Schrag 2021). As such, further investigation of the molecular mechanisms of CRC progression and exploration of new therapeutic strategies to ameliorate the prognosis of CRC patients are warranted.

Circular RNAs (circRNAs) are endogenous non-coding RNAs generated by variable splicing with high expression abundance, tissue specificity, and conservation (Patop et al. 2019; Yang et al. 2021). Recent years have witnessed a spurt of progress in genome sequencing, which has resulted in the discovery of numerous circRNAs. Further studies have clearly identified circRNAs as promising biomarkers in the tumor treatment process (Zhang et al. 2018b; Zhou et al. 2020b). In cancer development, circRNAs are engaged in diverse biological processes such as tumor growth, invasion, and metastasis (Li et al. 2020, 2022). For instance, circANXA2 augmented the malignant process of lung cancer cells via miR-33a-5p/PDPK1 network (Ju et al. 2021). CircNR3C2 was notably diminished in triple-negative breast cancer, which could perform as a tumor suppressor gene (Fan et al. 2021). Nevertheless, circRNAs as a large group of non-coding RNAs, only few of them with explicit biological function have been explored. There are still substantial circRNAs with tumor-promoting/suppressing properties to be further probed in CRC.

Ferroptosis is a newly defined iron-dependent programmed cell death accompanied by excessive accumulation of reactive oxygen species (ROS) and lipid peroxides (Jiang et al. 2021). In recent years, an increasing number of studies have linked ferroptosis to the development and treatment response of various malignancies (Chen et al. 2021), including CRC (Yan et al. 2023). Induction of ferroptosis provides novel strategies and possibilities for achieving inhibition of tumor progression (Yin et al. 2022). Notably, circRNA has been validated to be participated in modulating the process of ferroptosis, thereby affecting the tumorigenesis and advancement (Zuo et al. 2022). For example, circLRFN5 suppressed the growth and stemness of glioblastoma by PRRX2/GCH1-induced ferroptosis (Jiang et al. 2022). CircLMO1 retarded the proliferation and invasion of cervical cancer cells through regulating miR-4291/ACSL4-mediated ferroptosis (Ou et al. 2022). Nevertheless, further investigation is required to reveal and elucidate the regulatory mechanism of circRNA-mediated ferroptosis in CRC.

In this study, we identified a novel tumor suppressor and ferroptosis promoter circRNA (circ_0087851) in CRC. Importantly, we observed that circ_0087851 was substantially downregulated in CRC tissues and cells. Moreover, overexpression of circ_0087851 suppressed the malignant phenotype and promoted ferroptosis of CRC cells. Mechanistically, circ_0087851 performed its biological function by modulating the miR-593-3p/BAP1 axis. Overall, our present work disclosed that circ_0087851 might be an underlying biomarker and promising therapeutic target for CRC treatment.

## Materials and methods

### CRC tissue specimens

Thirty-one paired colorectal cancerous and paracancerous samples were obtained from patients at the Affiliated Hospital of Xiangnan University. The pathological diagnosis of colorectal cancer specimens was performed by two pathologists with specialized training. The extracted tissue specimens were promptly snap-frozen in liquid nitrogen and stored at − 80 °C. All experiments were authorized by the Affiliated Hospital of Xiangnan University Ethics Committee, and informed consent was obtained from all patients before surgery.

### Cell culture

Normal human colorectal epithelial cell line (NCM460) and four diverse CRC cell lines (HT29, SW480, HCT116, and RKO) were purchased from the American Type Culture Collection (ATCC, USA). The cell lines were grown in RPMI-1640 medium (Gibco, USA) containing 10% FBS (Beyotime Biotechnology, China) and 1% penicillin/streptomycin (Beyotime Biotechnology), followed by culturing in a thermostatic incubator (37 °C, 5% CO_2_).

### Cell transfection

Lipofectamine 3000 reagent (Invitrogen, USA) was used to transfect HCT116 and RKO cells with oligonucleotides according to the manufacturer’s protocols. Circ_0087851 small interfering RNA (si-circ_0087851) and negative control (si-NC), BAP1 small interfering RNA (si-BAP1) and its negative control (si-NC), miR-593-3p mimic/miR-593-3p inhibitor, and their corresponding negative control miR-NC mimic/anti-NC were provided by Anhui General Biological Company (China). CRC cells were transfected for 48 h, and transfection efficiency was validated by qRT-PCR for subsequent experiments. Besides, lentivirus-oe-circ_0087851 and corresponding control were purchased from Anhui General Biological Company (China), and lentiviral transfection was conducted on the basis of manufacturer’s protocols.

### qRT-PCR

Total RNA was extracted from the obtained tissues or cultured cells using TRIzol reagent (Beyotime Biotechnology). In accordance with the manufacturer’s protocols, reverse transcription was performed to synthesize cDNA using the PrimeScript^™^ RT Master Mix (Takara). Subsequently, SYBR ® Premix Ex Taq^™^ (Takara) was used for qRT-PCR. U6 and GAPDH were used as internal controls, and the relative expression of circ_0087851, miR-593-3p, and BAP1 was calculated using the 2^–∆∆Ct^ method. The primer sequences of the above genes were as follows (5′ → 3′):

circ_0087851-F: ACCATGCAGGTCACCCTGAAGA,

circ_0087851-R: GTGTGGACACTGCTTTGGGTTT,

miR-593-3p-F: AGAATCTGTCAGGCACCAGCC,

miR-593-3p-R: ACAAACCCAGCACCACTCCT,

BAP1-F: GACCCAGGCCTCTTCACC,

BAP1-R: AGTCCTTCATGCGACTCAGG,

GAPDH-F: TCAAGATCATCAGCAATGCC,

GAPDH-R: CGATACCAAAGTTGTCATGGA,

U6-F: GAGGCACAGCGGAACG,

U6-R: CTACCACATAGTCCAGG.

### Nuclear-cytoplasmic fractionation

For subcellular localization analysis, the PARIS Kit (Invitrogen) was used to separate nuclear and cytoplasmic RNAs from CRC cells, according to the manufacturer’s protocols. Subsequently, the expression of circ_0087851 in the nucleus and cytoplasm was quantified by qRT-PCR, with U6 and GAPDH as controls.

### Fluorescence in situ hybridization (FISH)

According to the manufacturer’s instructions, circ_0087851-specific probes were synthesized using Fish kit (GenePharma, Shanghai, China). After incubation with pre-hybridization solution, the CRC cells were treated with hybridization solution carrying probes against circ_0087851 at 42 °C overnight. Then, DAPI was employed for nuclear staining. Finally, the cell images were taken under a confocal laser scanning microscope.

### Treatment of RNase R

Total RNA samples (2 μg) were added to the control and experimental groups, and the experimental RNA was treated with 3 U/μg RNase R (Epicentre, Madison, USA) for 30 min at 37 °C. Next, circ_0087851 and linear mRNA expression were evaluated using qRT-PCR to assess the stability of circ_0087851.

### Colony formation assay

Well-growing HCT116 and RKO cells were collected and digested using 0.25% trypsin (Beyotime Biotechnology). Then, 500 cells were inoculated into six-well plates and cultured in a humidified incubator (37 °C, 5% CO_2_) for 2 weeks. The cells were then treated with PBS (Beyotime Biotechnology), fixed with 4% paraformaldehyde (Beyotime Biotechnology), and stained with 0.1% crystal violet (Beyotime Biotechnology). Finally, the colonies were counted under an Olympus microscope.

### CCK-8 assay

HCT116 and RKO cells were seeded in 96-well plates at a density of 2500 cells per well after transfection. Next, in accordance with the manufacturer’s protocols, 10 μL of diluted CCK-8 reagent (Beyotime Biotechnology) was added to each well at various times. The cells were then incubated for 3 h under light-free conditions. The absorbance of the cells was measured at 450 nm using a microplate reader. Moreover, the CRC cells were treated with DMSO (Solarbio, China), 5 µM/mL erastin (Selleck Chemicals, USA; ferroptosis activatior) and 2 µM/mL ferrostatin-1(Fer-1, Selleck Chemicals; ferroptosis inhibitor) for 24 h. Then cells were incubated CCK-8 reagent and cell viability was calculated through detecting the cell absorbance at 450 nm.

### Transwell assay

Transwells (Millipore, Billerica, MA, USA) were used to assess the migratory and invasive capabilities of CRC cells. Briefly, Matrigel (BD Biosciences, USA) was coated in the upper chambers for cell invasion detection, whereas no Matrigel was used for cell migration detection. Transfected HCT116 and RKO cells (2 × 10^4^/well) were inoculated into the upper chamber, and RPMI-1640 medium containing 10% FBS was added to the bottom chamber. The cells were then incubated for 24 h, followed by treating with 4% paraformaldehyde and staining with 0.1% crystal violet. Finally, migrated and invaded cells were counted under a microscope (Olympus, Japan).

### Measurement of iron, Fe^2+^, and lipid ROS levels

The intracellular iron, Fe^2+^, and lipid ROS of CRC cells were measured by adopting corresponding kits based on the manufacturer’s instructions. Iron Assay Kit (Sigma, USA, MAK025) was employed to detect the iron and Fe^2+^ levels. Briefly, 2 × 10^6^ transfected CRC cells were rapidly homogenized in 5 volumes of iron assay buffer, followed by centrifuging at 16,000* g* for 10 min at 4 °C to remove insoluble material. Then, 50 μL sample and 50 μL assay buffer were added to 96-well plate. To determine iron and Fe^2+^ levels, 5 μL iron reducer or 5 μL iron assay buffer was added to each well. Next, the samples were mixed under a horizontal shaker and incubated at 25 °C for 30 min away from light. Afterward, 100 μL iron probe was added to the mixture and incubated for another 1 h at 25 °C. Finally, the absorbance was detected at 593 nm through a microplate reader. For detecting intracellular ROS levels, 2′,7′-dichlorofluorescin diacetate (DCFH-DA; Beyotime, S0033) staining was adopted. Transfected CRC cells were harvested and washed with PBS. Then DCFH-DA diluted to a final concentration of 10 μmol/L was added and reacted for 30 min at 37 °C away from light. After three times of PBS cleaning, DAPI was used to stain nuclei. At last, the images were observed and photographed under fluorescence microscope (Nikon, Eclipse Ci-L) in time, and ROS levels were measured by Image J software.

### Luciferase reporter assay

To identify the relationship between circ_0087851 and miR-593-3p, together with miR-593-3p and BAP1, first, the wild-type (WT) or mutant (MUT) circ_0087851 sequence carrying the miR-593-3p binding sites was inserted into the pmirGLO vector (Promega, USA) to construct circ_0087851 WT and circ_0087851 MUT. Similarly, luciferase reporter vectors carrying 3′-UTR wild-type fragments or mutant type fragments of BAP1 mRNA were obtained and termed BAP1 WT and BAP1 MUT. Next, the constructed reporter vectors and miR-593-3p mimics or their negative controls were co-transfected into HCT116 and RKO cells. After 2 days, the luciferase reporter gene assay system (Promega, USA) was used to quantify the corresponding luciferase activity.

### Biotin-labeled RNA pulldown

The BersinBioTM RNA pulldown kit (BersinBio, China) was used for RNA pulldown analysis. Biotin-labeled oligonucleotide probes targeting miR-593-3p were synthesized and termed biotin–miR-593-3p, with biotin-NC serving as the negative control probe. Then, the probe was incubated with CRC cell lysate supernatant and streptavidin magnetic beads (Invitrogen) at 4 °C to collect probe–bead complexes. Finally, qRT-PCR was performed to evaluate the bound RNA.

### Western blotting

CRC cells and tissues were lysed via RIPA buffer (Thermo Fisher). Subsequently, a BCA protein assay kit (Millipore) was applied for protein concentration measurement based on the manufacturer’s protocols. The collected proteins were separated by SDS-PAGE (GenScript) and transferred onto polyvinylidene fluoride (PVDF) membranes (Millipore). The membranes were blocked with nonfat milk for 1 h and interacted with dilutions of primary antibodies overnight at 4 °C. The membranes were then treated with proper horseradish peroxidase-labeled secondary antibodies. Finally, all the targeted bands were observed using an enhanced chemiluminescence kit (Thermo Fisher). Primary antibodies against BAP1 (ab245391, 1:2000) and GAPDH (ab59164, 1:2000) were purchased from Abcam (Cambridge, UK).

### Immunohistochemistry (IHC)

Freshly excised tissues were immobilized in 4% paraformaldehyde, embedded in paraffin, and sectioned. The sections were dewaxed with xylene, dehydrated with anhydrous alcohol, and blocked with endogenous peroxidase with 3% H_2_O_2_. The sections were then placed in citrate buffer for microwave antigen repair. Sections were incubated with anti-BAP1 primary antibody incubated overnight at 4 °C. Afterward, the collected sections were rinsed two to three times with PBS and then incubated with HRP-conjugated secondary antibody for 1 h at 37 °C. The number of BAP1-positive cells was measured using a microscope (Nikon, Japan) after staining with DAB (Beyotime).

### Animal assay

Female BALB/c nude mice (6–8 weeks old) were obtained from Vital River (Beijing, China). All animal experiments were performed according to the institutional protocol and authorized by the Affiliated Hospital of Xiangnan University Ethics Committee. The experimental nude mice were randomly allocated to the oe-circ and oe-NC groups (*n* = 3 for each group). CRC cells (2 × 10^6^/mL) stably transfected with the circ_0087851 overexpression vector (oe-circ) and empty vector (oe-NC) were injected into the mice. Later, tumor weight and volume (0.5 × length × width^2^) were monitored and quantified every 7 days until day 28. Finally, the mice were killed and the tumors were excised, weighed, and imaged.

### Statistical analysis

Statistical analysis of the experimental results was performed using GraphPad Prism 8.0 software (La Jolla, CA, USA). Each experiment was independently conducted at least three times, and the data collected are displayed as the mean ± SD. Pearson correlation analysis was used to analyze the correlation between circ_0087851 and miR-593-3p expression. Student’s *t* test was applied to assess statistical differences. *P* < 0.05 was considered statistically significant.

## Circ_0087851 was notably diminished in CRC

**Fig. 1:**
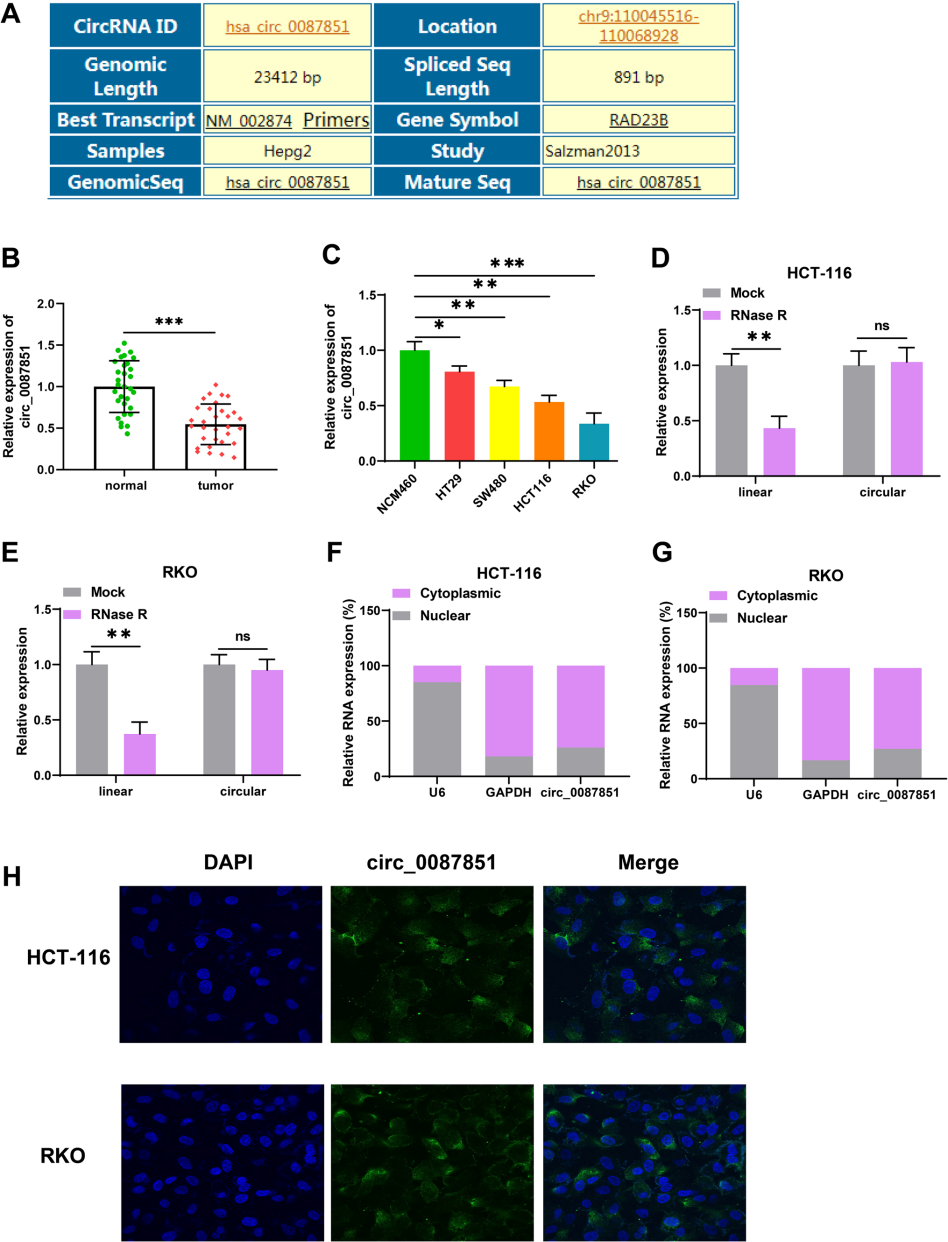
Circ_0087851 was notably diminished in CRC. **A** The genomic information of circ_0087851. **B** Circ_0087851 expression was validated in 31 pairs of CRC tissues and paracancerous tissues via qRT-PCR. **C** qRT-PCR was performed to assess the differential expression of circ_0087851 within diverse cell lines (NCM460, HT29, SW480, HCT116, and RKO). **D**, **E** Circ_0087851 and linear mRNA expression were detected utilizing qRT-PCR. **F**, **G** qRT-PCR was conducted to analyze the subcellular localization of circ_0087851 in the cytoplasm and nuclear of CRC cells. **H** FISH assays exhibited the cellular localization of circ_0087851 in CRC cells. *P < 0.05, **P < 0.01, ***P < 0.001

First, we analyzed the genomic information of circ_0087851 through Circinteractome database (https://circinteractome.nia.nih.gov/). Circ_0087851 was derived from the RAD23B gene and located in chr9: 110045516–110068928 (Fig. 1A). To validate the expression pattern of circ_0087851 in CRC, we examined the circ_0087851 expression in CRC tissues and cells using qRT-PCR. As displayed in Fig. 1B, C, circ_0087851 levels were notably diminished in 31 pairs of CRC tissues. Meanwhile, clinicopathological features showed that aberrant circ_0087851 expression was associated with tumor size, lymph metastasis, histopathological grade, and TNM stage in CRC patients (Table 1). In addition, circ_0087851 was apparently downregulated in four CRC cell lines (HT29, SW480, HCT116, and RKO) compared to normal colorectal endothelial cell lines (NCM460). Subsequently, we evaluated the stability of the circ_0087851. After treatment with RNase R, circ_0087851 was more stable than the linear mRNA in HCT116 and RKO cells (Fig. 1D and E). Next, we confirmed the subcellular localization of circ_0087851 in CRC cells. The qRT-PCR results presented that circ_0087851 primarily existed in the cytoplasm of HCT116 and RKO cells (Fig. 1F and G). Moreover, fish assays also exhibited that circ_0087851 was mainly localized to the cytoplasm in CRC cells (Fig. 1H). Collectively, the above analysis demonstrated that circ_0087851 was substantially decreased in CRC, and it might be a potential tumor suppressor in CRC progression.

**Table 1:** The relationship between circ_0087851 expression and clinicopathological features in CRC tissues

	circ_0087851 expression		*P* value (Chi-square test)
	Low (*n* = 16)	High (*n* = 15)	
Age (years)			
≤ 50	7	8	0.5936
> 50	9	7	
Gender			
Male	8	9	0.5761
Female	8	6	
Tumor size (cm)			
≤ 5	5	11	0.0191*
> 5	11	4	
Location			
Colon	10	6	0.2103
Rectum	6	9	
Lymph metastasis			
No	5	12	0.0064*
Yes	11	3	
Histopathological grade			
Well–moderate	6	11	0.0451*
Poor	10	4	
TNM stage			
I + II	4	10	0.0198*
III + IV	12	5	

### Overexpression of circ_0087851 restricted cell viability, migration, and invasion and facilitated ferroptosis in CRC cells

**Fig. 2:**
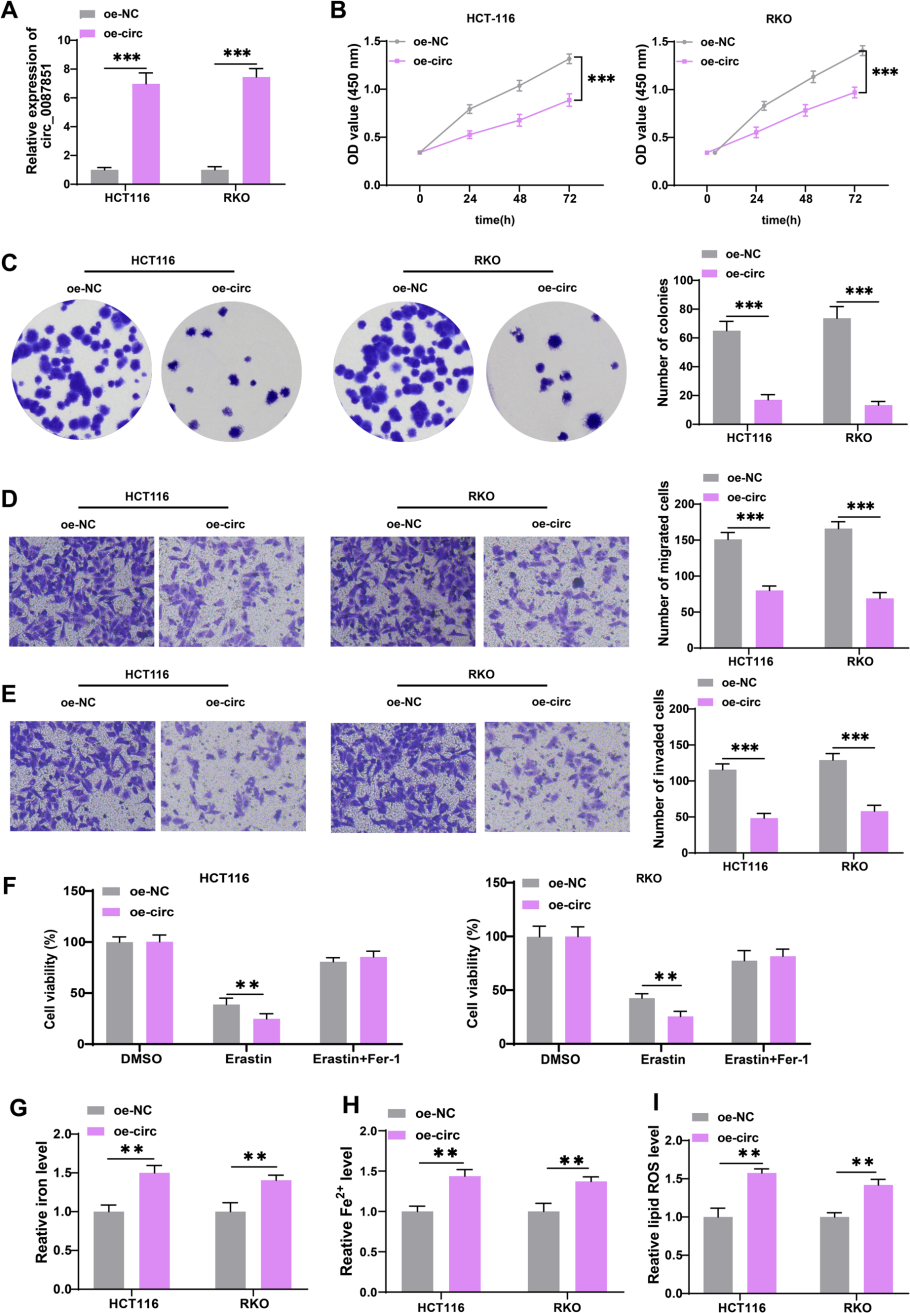
Overexpression of circ_0087851 restricted cell viability, migration and invasion and facilitated ferroptosis in CRC cells. HCT116 and RKO cells were transfected with oe-NC or oe-circ_0087851. **A** qRT-PCR was conducted to validate the transfection efficiency in CRC cells. **B** CCK-8 was performed to assess the viability of CRC cells after transfection. **C** Colony formation was conducted to evaluate the proliferation of CRC cells after transfection. **D**, **E** Transwell assays were employed to monitor the migration (**D**) and invasion (**E**) of CRC cells after transfection. **F** CCK-8 was used to analyze the cell viability of transfected cells after treated with DMSO, erastin or ferrostatin-1. **G**–**I** The levels of iron (G), Fe^2+^ (**H**) and lipid ROS (**I**) of CRC cells were measured after transfection. **P < 0.01, ***P < 0.001

Considering that circ_0087851 was downregulated in CRC, we overexpressed circ_0087851 to further explore its biological role in CRC progression. First, an overexpression vector of circ_0087851 was transfected into HCT116 and RKO cells, which successfully achieved high overexpression efficiency (Fig. 2A). Then we performed CCK-8 assays to assess the viability of CRC cells after transfection. The results displayed that circ_0087851 overexpression potently diminished the growth viability of HCT116 and RKO cells (Fig. 2B). Similarly, the colony formation assay revealed that the introduction of circ_0087851 mitigated the proliferative capacity of CRC cells (Fig. 2C). Next, transwell assays indicated that the insertion of circ_0087851 substantially restricted the migration and invasion processes of CRC cells (Fig. 2D and E). Emerging studies have disclosed that ferroptosis is closely linked to tumor progression. Here, we evaluated the impacts of circ_0087851 on ferroptosis of CRC cells. After cells were treated with ferrostatin-1 (2 µM) or erastin (5 µM) for 24 h, we assessed the change of cell viability. Interestingly, we found that overexpression of circ_0087851 obviously reinforced the erastin (ferroptosis activatior)-induced viability inhibition, while this process was reversed by ferrostatin-1 (ferroptosis inhibitor) in CRC cells (Fig. 2F). The results implied that circ_0087851 upregulation suppressed cell viability via a ferroptosis-dependent manner. Moreover, we observed that circ_0087851 overexpression elevated the intracellular iron and Fe^2+^ levels (Fig. 2G and H). Furthermore, introduction of circ_0087851 also triggered the accumulation of lipid ROS in CRC cells (Fig. 2I and Figure S1). These findings revealed that circ_0087851 overexpression suppressed cell growth and metastasis and augmented ferroptosis in CRC.

### Circ_0087851 sponged miR-593-3p in CRC cells

**Fig. 3:**
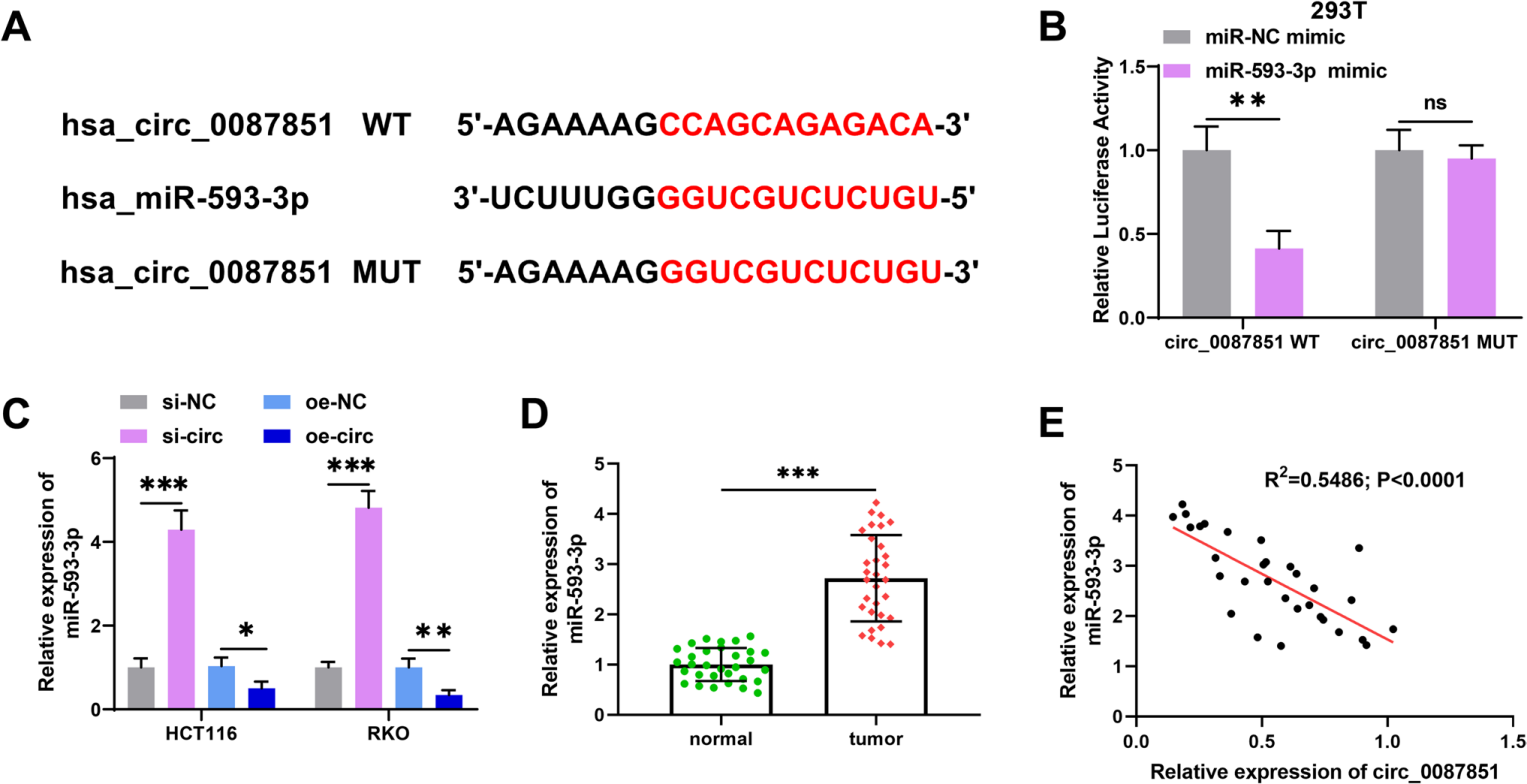
Circ_0087851 sponged miR-593-3p in CRC cells. **A** The binding sites between circ_0087851 and miR-593-3p was predicted using Circinteractome database. **B** The combination of circ_0087851 and miR-593-3p was verified through luciferase assay. **C** The expression of miR-593-3p was examined in HCT116 and RKO cells after circ_0087851 knockdown or overexpression. **D** qRT-PCR was adopted to validate miR-593-3p expression in CRC patients (*n* = 31). **E** Correlation analysis between the expression of circ_0087851 and miR-593-3p. *P < 0.05, **P < 0.01, ***P < 0.001

To further probe the underlying mechanism of circ_0087851-mediated CRC progression, we used Circinteractome online database (https://circinteractome.nia.nih.gov/) to identify the downstream target miRNAs of circ_0087851. Here, we found that miR-593-3p had complementary binding sites to circ_0087851 (Fig. 3A). Subsequently, luciferase reporter assays were conducted to validate the presumed binding between circ_0087851 and miR-593-3p. We observed that the luciferase activity of the circ_0087851 wild type was diminished by the introduction of miR-593-3p mimic compared to mimic NC, whereas it did not affect the circ_0087851 mutant by miR-593-3p mimic transfection (Fig. 3B). Then we applied qRT-PCR to further elucidate the regulatory relationship between circ_0087851 and miR-593-3p. The data revealed that silencing of circ_0087851 substantially enhanced miR-593-3p levels in CRC cells, while circ_0087851 overexpression functioned oppositely (Fig. 3C). Moreover, qRT-PCR presented that miR-593-3p was substantially elevated in CRC tissues compared to paired paracancerous tissues (Fig. 3D). In addition, miR-593-3p expression was inversely correlated with circ_0087851 (Fig. 3E). Taken together, these data suggested that circ_0087851 conversely modulated miR-593-3p in CRC cells.

### MiR-593-3p targeted BAP1 in CRC cells

**Fig. 4:**
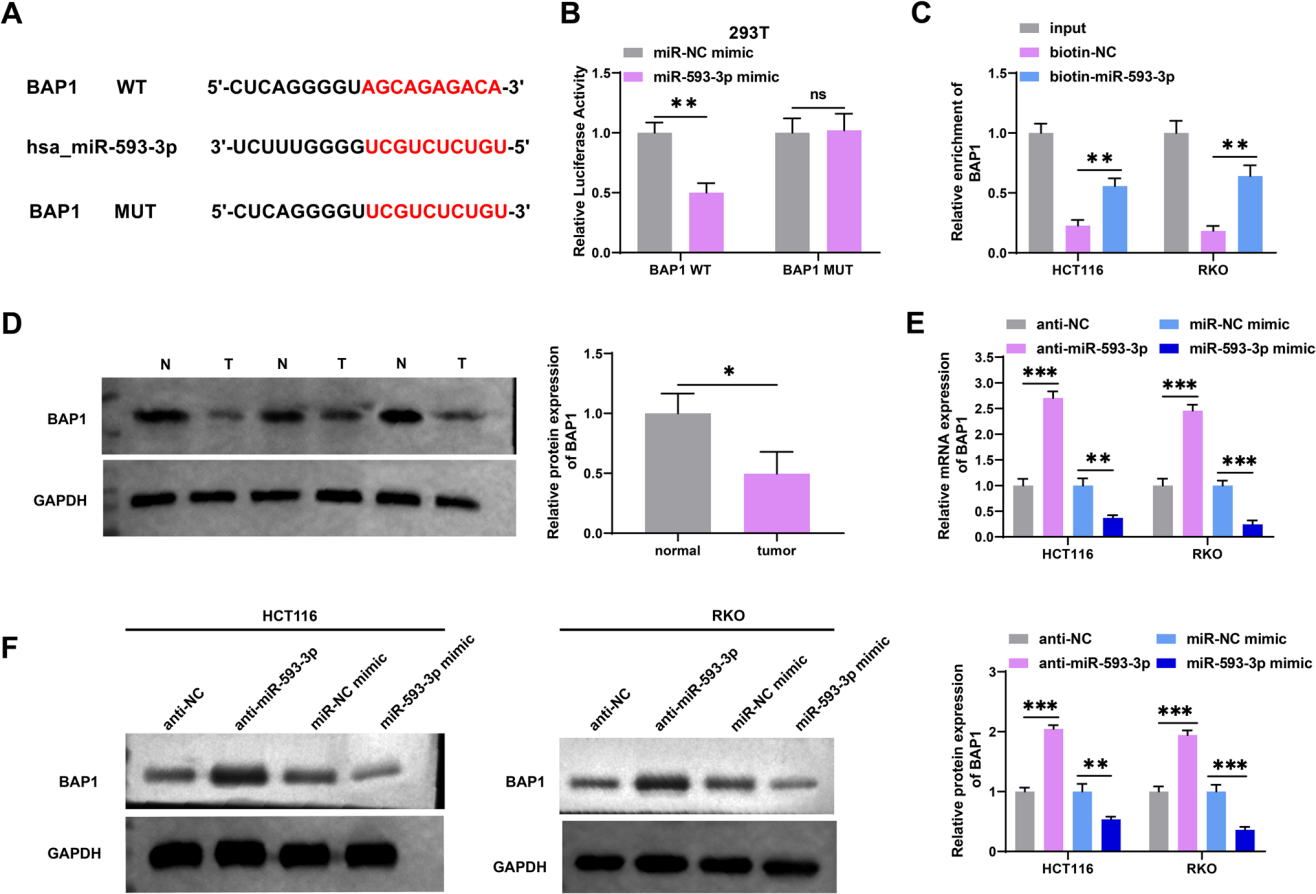
MiR-593-3p targeted BAP1 in CRC cells. **A** The combinative sites of BAP1 3′‐UTR and miR-593-3p were predicted using TargetScan database. **B** Luciferase reporter assay was employed to confirm the interaction of BAP1 and miR-593-3p. **C** The direct binding of miR-593-3p to BAP1 was validated using RNA pulldown analysis. **D** BAP1 protein expression in CRC patients (*n* = 3). The BAP1 mRNA (**E**) and protein (**F**) expression were examined in CRC cells transfected with anti-NC, miR-NC mimic, anti-miR-593-3p or miR-593-3p mimic. *P < 0.05, **P < 0.01, ***P < 0.001

Next, we performed bioinformatic analysis to probe the downstream target of miR-593-3p. Based on the Targetscan database (https://www.targetscan.org/vert_80/), BAP1 was predicted as the candidate target of miR-593-3p. Figure 4A displayed that there were binding sites between the 3′-UTR of BAP1 and miR-593-3p. Afterward, we employed luciferase reporter and RNA pulldown assays to confirm the binding relationship between BAP1 and miR-593-3p. In 293 T cells, reduced luciferase activity was detected for BAP1-WT with the introduction of the miR-593-3p mimic relative to the control group, whereas that of BAP1-MUT showed no apparent change (Fig. 4B). Similarly, the data from the RNA pulldown experiments further validated the interaction between BAP1 and miR-593-3p (Fig. 4C). Moreover, we observed that BAP1 protein expression was substantially attenuated in CRC tissues (Fig. 4D). Furthermore, qRT-PCR and western blotting revealed that BAP1 mRNA and protein levels were notably enhanced in HCT116 and RKO cells by miR-593-3p inhibitor transfection, whereas miR-593-3p mimic apparently restrained BAP1 expression (Fig. 4E and F). Collectively, these results disclosed that miR-593-3p directly and inversely targeted BAP1 in CRC.

### MiR-593-3p overexpression or BAP1 knockdown reversed the effect of circ_0087851-mediated growth, metastasis, and ferroptosis in CRC cells

**Fig. 5:**
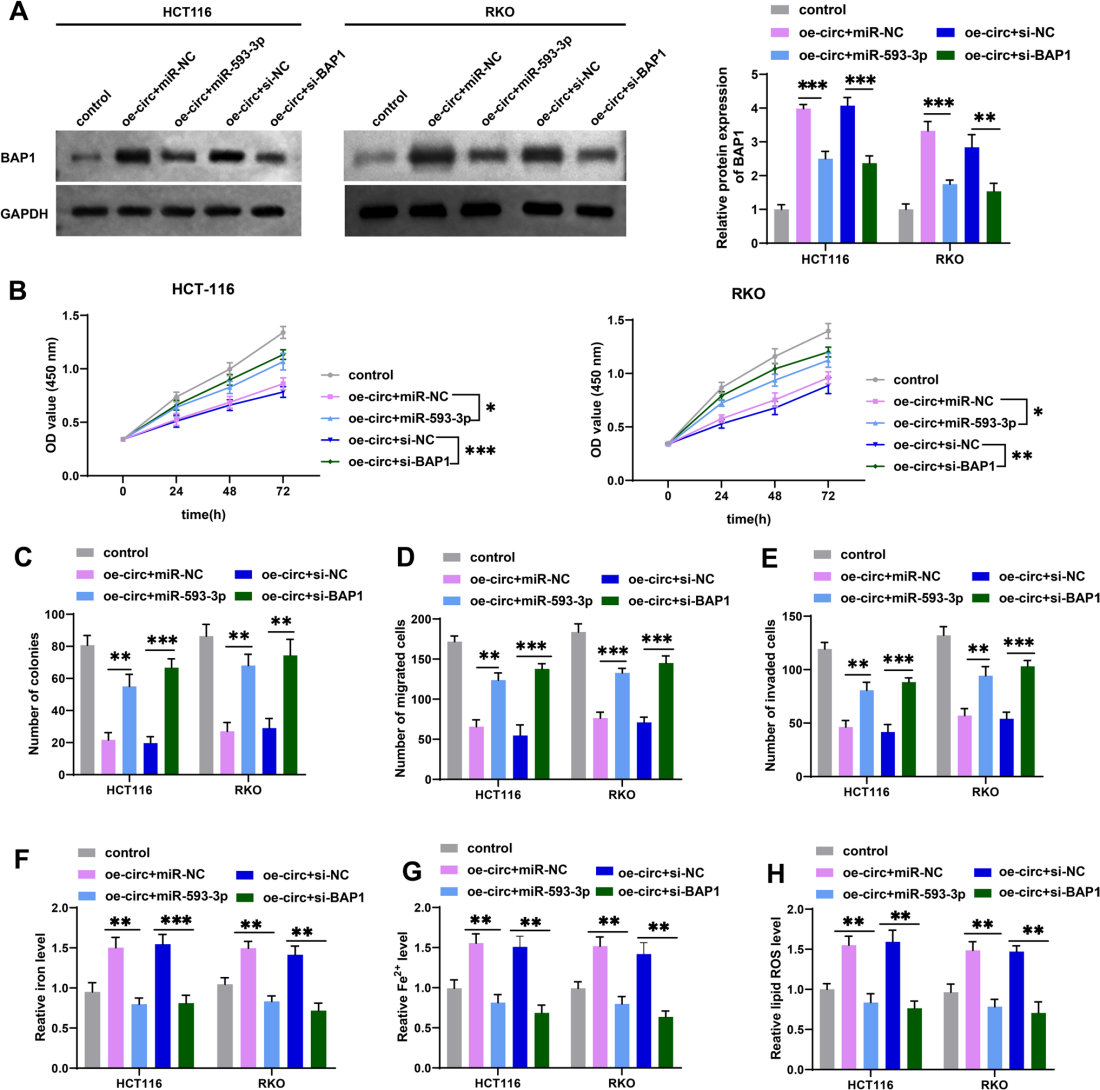
MiR-593-3p overexpression or BAP1 knockdown reversed the effect of circ_0087851-mediated growth, metastasis, and ferroptosis in CRC cells. HCT116 and RKO cells were transfected with oe-circ + miR-NC, oe-circ + miR-593-3p, oe-circ + si-NC, or oe-circ + si-BAP1. **A** BAP1 protein levels in CRC cells were validated through western blotting after different treatments. **B** Cell viability was examined through CCK-8 after different treatments. **C** The cloning ability of CRC cells was assessed through cell colony formation after different treatments. **D**, **E** The cell migration (**D**) and invasion (**E**) of CRC cells were identified through transwell assay after different treatments. **F**–**H** The changes of intracellular iron (F), Fe^2+^ (**G**) and lipid ROS (**H**) of CRC cells were evaluated after different treatments. *P < 0.05, **P < 0.01, ***P < 0.001

The above findings revealed a potential circ_0087851/miR-593-3p/BAP1 regulatory network in CRC, so we conducted rescue experiments to identify whether circ_0087851 targeted miR-593-3p/BAP1 axis and restrained the malignant process of CRC cells. We first constructed HCT116 and RKO cells transfected with oe-circ _0087851 + miR-NC, oe-circ_0087851 + miR-593-3p, oe-circ_0087851 + si-NC, and oe-circ_0087851 + si-BAP1. Then the protein expression of BAP1 was assessed using western blotting. As shown in Fig. 5A, circ_0087851 overexpression notably elevated the BAP1 protein levels, whereas miR-593-3p introduction or silencing of BAP1 reversed this effect. Moreover, CCK-8 and colony formation experiments showed that upregulation of circ_0087851 diminished CRC cell viability and growth, whereas miR-593-3p overexpression or BAP1 knockdown partially reversed this phenomenon (Fig. 5B and C). Likewise, transwell assays exhibited that the migratory and invasive capabilities of CRC cells were attenuated in the presence of oe-circ_0087851, while these inhibitory effects were successfully blocked by miR-593-3p overexpression or BAP1 inhibition (Fig. 5D and E). Additionally, we evaluated the role of circ_0087851/miR-593-3p/BAP1 axis in modulating CRC cell ferroptosis. Notably, miR-593-3p mimic or BAP1 silencing rescued the mitigated iron, Fe^2+^, and lipid ROS levels induced by circ_0087851 overexpression in CRC cells (Fig. 5F–H). Overall, these results illustrated that circ_0087851 suppressed CRC cell malignant phenotypes and augmented ferroptosis of CRC cells through the miR-593-3p/BAP1 axis.

### Overexpressed circ_0087851 restrained tumor growth of CRC in vivo

**Fig. 6:**
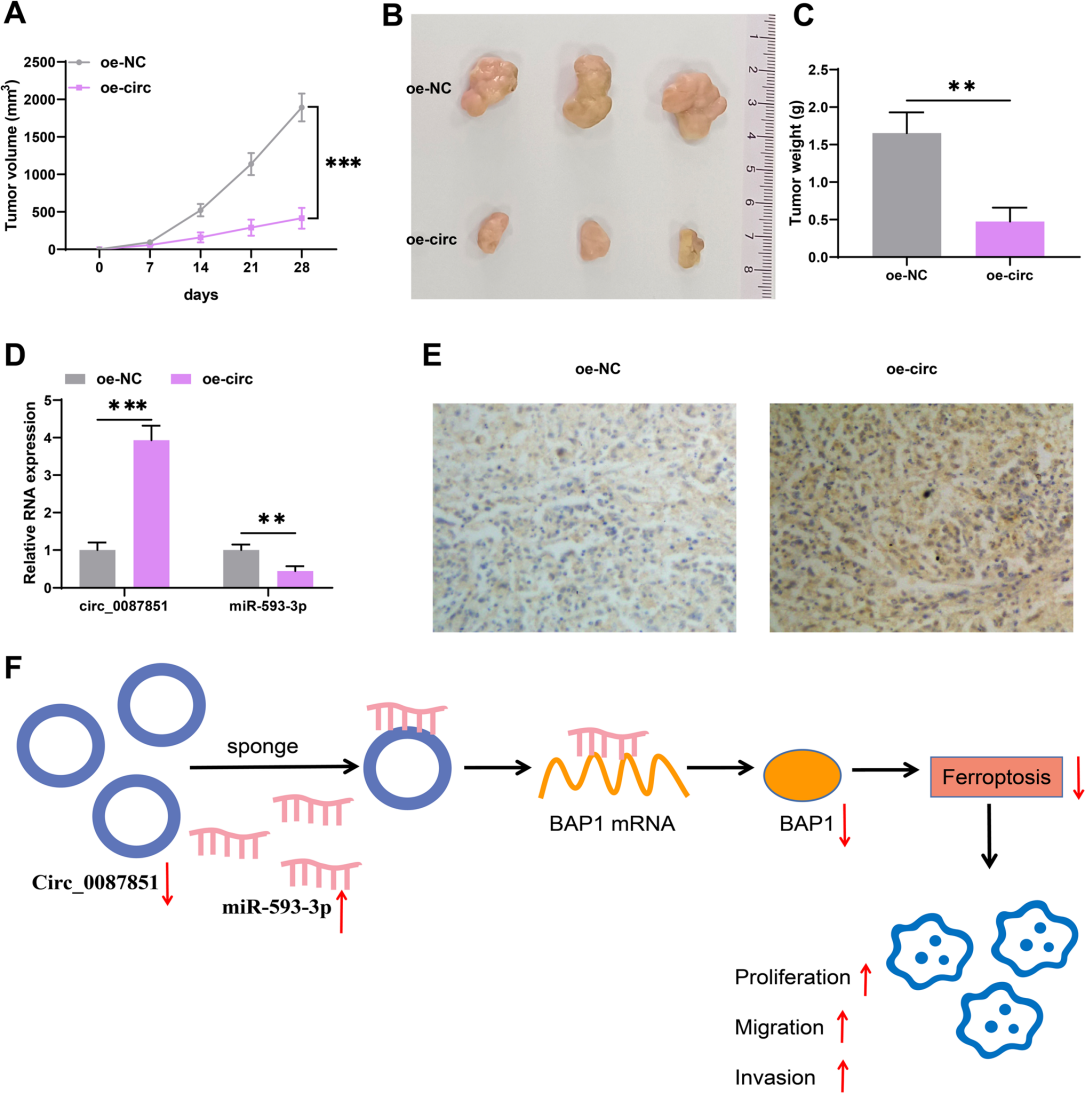
Silenced circ_0087851 restrained tumor formation of CRC in vivo. **A** The detection of tumor volume every 7 days after injection. **B** Representative image of tumor progression. **C** Tumor weight was monitored. **D** The expression of circ_0087851 and miR-593-3p in xenograft tumor tissues. **E** BAP1 protein levels in xenograft tumors were assessed through IHC staining. **F** Schematic diagram displayed the mechanism of circ_0087851-regulated ferroptosis and progression of CRC. **P < 0.01, ***P < 0.001

Based on the above results, we performed animal experiments to further validate the anti-cancer effect of circ_0087851 in vivo. We constructed the xenograft tumor model in BALB/c nude mice using HCT116 cells with stable overexpression of circ_0087851, and designed two experimental groups: oe-NC group and oe-circ_0087851 gruop. 28 days later, we observed that tumor volume and weight were attenuated in the circ_0087851 overexpression group relative to the control group (Fig. 6A–C). Moreover, we assessed the expression of circ_0087851 and miR-593-3p in the tumor tissues of the mice. As expected, circ_0087851 expression was elevated in the oe-circ_0087851 group, whereas miR-593-3p expression was diminished by the circ_0087851 upregulation (Fig. 6D). In addition, IHC staining showed an increase in BAP1-positive cells in the circ_0087851 overexpression group (Fig. 6E). Taken together, our results revealed that circ_0087851 restricted CRC growth in vivo.

## Discussion

As a common tumor of the digestive system, CRC is a major global public health challenge. Its therapeutic effect is generally undesirable due to high insidiousness and strong aggressiveness. Thus, it is urgent to explore accurate and effective molecular targets to enhance therapeutic benefits and ameliorate patient prognosis. Here, we presented an interesting molecular mechanism in regulating CRC advancement. Circ_0087851 facilitated ferroptosis of CRC cells and restrained CRC progression through targeting miR-593-3p/BAP1 network (Fig. 6F).

CircRNAs has been focusing much attention in the field of oncology due to their highly conserved phenotype and tissue specificity. In particular, emerging reports have demonstrated that circRNAs are closely linked to CRC progression and that the regulation of circRNAs can intervene with the malignant phenotype of CRC. Circ CAMSAP1 augmented cell viability in CRC by sponging miR-328-5p (Zhou et al. 2020a). CircHERC4 aggravates CRC metastasis by interacting with CTBP2/E-cadherin and sponging miR-556-5p (He et al. 2021). CircRNA_102049 expression was notably enhanced in CRC cells and augmented their viability (Zhu et al. 2022). Hence, searching for CRC-related circRNAs contributes to understanding the pathological mechanism of CRC and offering potential molecular targets for CRC clinical treatment. In this study, we identified a novel circRNA, circ_0087851, was markedly diminished in CRC tissues and cells. Based on the Circinteractome database, we found that hsa_circ_0087851 is derived from RAD23 homolog B (RAD23B) gene with a spliced sequence of 891 bp. Then we validated the biological function of circ_0087851 in CRC. Notably, we observed that overexpression of circ_0087851 hindered the growth, migration, and invasion capabilities of CRC cells in vivo, indicating that circ_0087851 functioned as a tumor suppressor in CRC.

Ferroptosis attracted our attention as a novel cell death mechanism. The resistance of tumor cells to cell death is a crucial obstacle in tumor treatment (Tong et al. 2022), and ferroptosis is a regulatory method of cell death caused by lipid peroxidation (Mbah and Lyssiotis 2022). Triggering ferroptosis can effectively augment the sensitivity of tumor cells to cell death, promote tumor suppression and attenuate cancer development, thereby improving patient treatment effects. To date, extensive studies have validated the circRNAs serve as key regulators of cell ferroptosis (Zuo et al. 2022). Here, we examined whether circ_0087851 is implicated in the ferroptosis of CRC cells. Interestingly, we observed that circ_0087851 overexpression triggered ferroptosis of CRC cells, as evidenced by the enhancement of intracellular iron, Fe^2+^, and ROS levels. The findings implied that circ_0087851 might be an advantageous target for suppressing CRC by modulating ferroptosis.

Importantly, numerous studies have highlighted that circRNAs can regulate the progression of tumor cells by sponging miRNAs and activating the circRNA–miRNA–mRNA axis (Rong et al. 2017; Yang et al. 2022). Based on the online Circinteractome database, miR-593-3p was predicted as the downstream target miRNA of circ_0087851. Previous studies have reported that miR-593-3p performed different roles in multiple tumors. Huang et al. proposed that miR-593-3p introduction propelled cell viability and colony formation in prostate cancer (Huang et al. 2021). Hata et al. revealed that miR-593-3p was remarkably elevated in peritoneal lavage fluid specimens collected from patients with pancreatic cancer (Hata et al. 2021). In contrast, miR-593-3p overexpression inhibits the malignant phenotype of breast cancer by targeting FGFR3 (Xie et al. 2020). Additionally, miR-593-3p suppressed the growth of gastric cancer, suggesting that miR-593-3p could be an anti-tumor gene for gastric cancer therapy (Dong et al. 2018). However, the role of miR-593-3p in CRC remains unclear. Our study validated that miR-593-3p was enhanced in CRC and inversely correlated with circ_0087851 expression. Furthermore, miR-593-3p overexpression partially abolished the inhibitory effects of circ_0087851 overexpression on viability, migration, and invasion of CRC cells. Meanwhile, introduction of miR-593-3p reversed the circ_0087851-mediated ferroptosis. Taken together, our data disclosed that circ_0087851 exerted its tumor-suppressive function by restricting miR-593-3p levels in CRC.

Further bioinformatic analysis validated BAP1 as a downstream target of miR-593-3p. Following previous work, BAP1 functioned as a significant tumor suppressor in human cancers (Masclef et al. 2021). BAP1 restricted the growth cycle of intrahepatic cholangiocarcinoma by regulating ERK1/2 and JNK/c-Jun axis (Chen et al. 2018). BAP1 restrained the malignant progression through activating the Hippo tumor suppressor pathway in pancreatic cancer (Lee et al. 2020). In CRC-related studies, low BAP1 expression was indicated to be associated with dismal prognosis in CRC patients (Tang et al. 2013). Here, we observed that BAP1 expression was significantly diminished in CRC tissues, which is consistent with the prior report. Notably, BAP1 was also identified as a ferroptosis-related gene, which could suppress tumor progression through triggering ferroptosis (Zhang et al. 2018a). BAP1 could restrain cystine uptake by inhibiting SLC7A11 expression, resulting in enhanced lipid peroxidation and ferroptosis (Zhang et al. 2018a). In this study, we expanded insights into how BAP1 affected CRC. The data obtained from the rescue experiments revealed that BAP1 expression was elevated after miR-593-3p knockdown or circ_0087851 overexpression. Additionally, silencing of BAP1 could reverse circ_0087851-induced tumor suppression and ferroptosis enhancement. The findings disclosed that circ_0087851 upregulation elevated the expression of BAP1 by sponging miR-593-3p, thereby facilitating ferroptosis and hindering CRC progression.

## Conclusions

In summary, our study revealed that circ_0087851 is downregulated in CRC. Overexpression of circ_0087851 suppressed CRC growth and metastasis by promoting miR-593-3p/BAP1-mediated ferroptosis. These findings suggested that circ_0087851 might be an effective target for ameliorating CRC treatment by regulating ferroptosis.

## Supplementary Information

Below is the link to the electronic supplementary material.

Supplementary file1 (DOCX 1613 KB)

## Data Availability

Data used to support the findings of this study are available from the corresponding author upon request.
