# Supplementary material for: Circ_0087851 suppresses colorectal cancer malignant progression through triggering miR-593-3p/BAP1-mediated ferroptosis
Source: J Cancer Res Clin Oncol. 2024 Apr 20;150(4):204. doi: 10.1007/s00432-024-05643-3 (PMC11032280; doi:10.1007/s00432-024-05643-3)
Supplement: Supplementary file 1 — Supplementary file1 (DOCX 1613 KB) [file 432_2024_5643_MOESM1_ESM.docx]

Supplementary Material


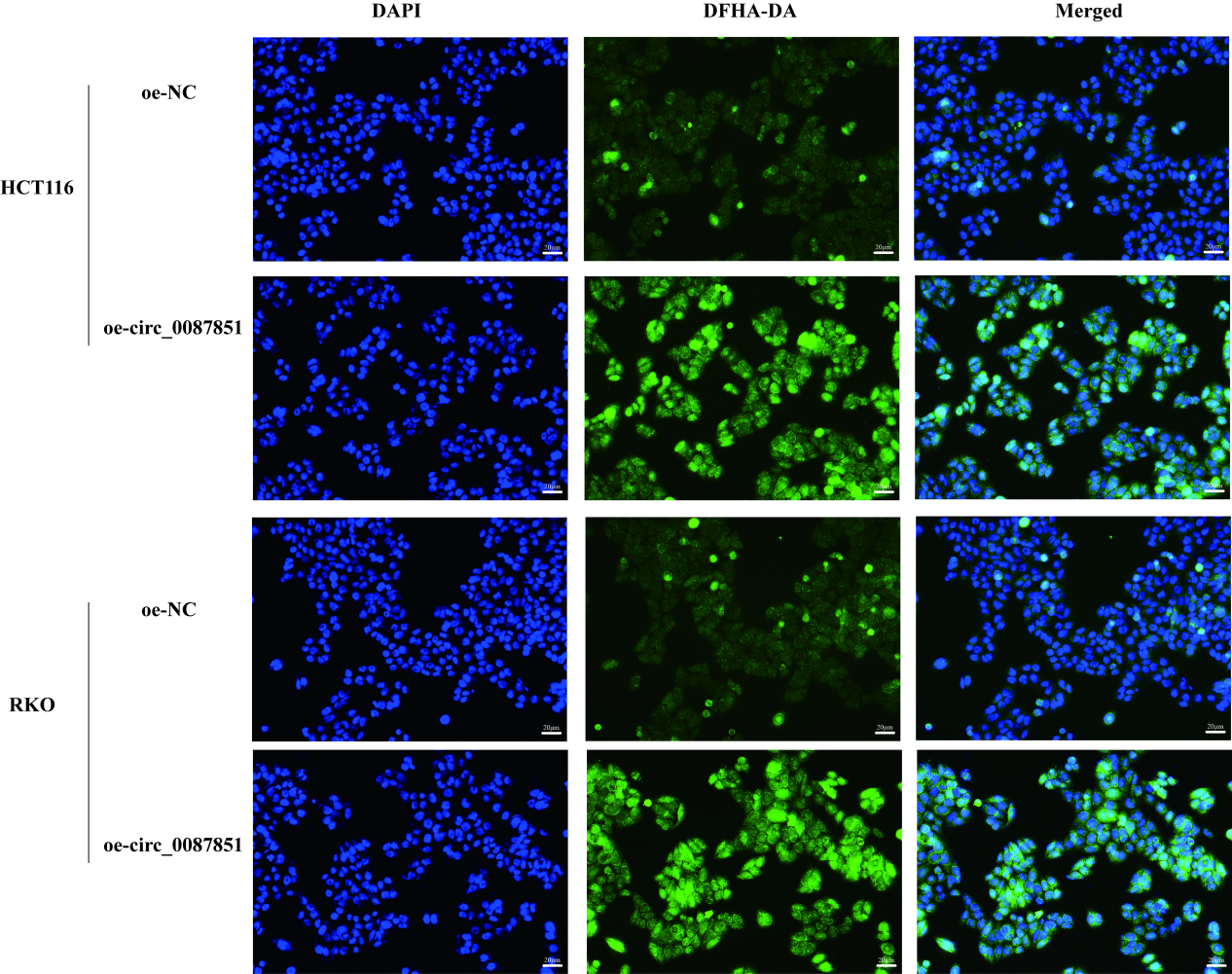


**Figure S1**: HCT116 and RKO cells were transfected with oe-NC or oe-circ_0087851, then ROS fluorescence staining was performed using 2′,7′‐dichlorodihydrofluorescein diacetate (DCFH‐DA) to visualize the intracellular ROS levels.
